# Identification of novel androgen receptor target genes in prostate cancer

**DOI:** 10.1186/1476-4598-6-39

**Published:** 2007-06-06

**Authors:** Unnati Jariwala, Jennifer Prescott, Li Jia, Artem Barski, Steve Pregizer, Jon P Cogan, Armin Arasheben, Wayne D Tilley, Howard I Scher, William L Gerald, Grant Buchanan, Gerhard A Coetzee, Baruch Frenkel

**Affiliations:** 1 Department of Biochemistry and Molecular Biology, Keck School of Medicine, University of Southern California, Los Angeles, USA; 2 Department of Preventive Medicine, Keck School of Medicine, University of Southern California, Los Angeles, USA; 3 Department of Urology, Keck School of Medicine, University of Southern California, Los Angeles, USA; 4 Department of Orthopedic Surgery, Keck School of Medicine, University of Southern California, Los Angeles, USA; 5 Dame Roma Mitchell Cancer Research Laboratories, School of Medicine, The University of Adelaide/Hanson Institute, Adelaide, Australia; 6 Genitourinary Oncology Service, Division of Solid Tumor Oncology, Memorial Sloan-Kettering Cancer Center, Department of Medicine, Joan and Sanford I. Weill College of Medicine, New York, NY, USA

## Abstract

**Background:**

The androgen receptor (AR) plays critical roles in both androgen-dependent and castrate-resistant prostate cancer (PCa). However, little is known about AR target genes that mediate the receptor's roles in disease progression.

**Results:**

Using Chromatin Immunoprecipitation (ChIP) Display, we discovered 19 novel loci occupied by the AR in castrate resistant C4-2B PCa cells. Only four of the 19 AR-occupied regions were within 10-kb 5'-flanking regulatory sequences. Three were located up to 4-kb 3' of the nearest gene, eight were intragenic and four were in gene deserts. Whereas the AR occupied the same loci in C4-2B (castrate resistant) and LNCaP (androgen-dependent) PCa cells, differences between the two cell lines were observed in the response of nearby genes to androgens. Among the genes strongly stimulated by DHT in C4-2B cells – D-dopachrome tautomerase (DDT), Protein kinase C delta (PRKCD), Glutathione S- transferase theta 2 (GSTT2), Transient receptor potential cation channel subfamily V member 3 (TRPV3), and Pyrroline-5-carboxylate reductase 1 (PYCR1) – most were less strongly or hardly stimulated in LNCaP cells. Another AR target gene, ornithine aminotransferase (OAT), was AR-stimulated in a ligand-independent manner, since it was repressed by AR siRNA knockdown, but not stimulated by DHT. We also present evidence for *in vivo *AR-mediated regulation of several genes identified by ChIP Display. For example, PRKCD and PYCR1, which may contribute to PCa cell growth and survival, are expressed in PCa biopsies from primary tumors before and after ablation and in metastatic lesions in a manner consistent with AR-mediated stimulation.

**Conclusion:**

AR genomic occupancy is similar between LNCaP and C4-2B cells and is not biased towards 5' gene flanking sequences. The AR transcriptionally regulates less than half the genes nearby AR-occupied regions, usually but not always, in a ligand-dependent manner. Most are stimulated and a few are repressed. In general, response is stronger in C4-2B compared to LNCaP cells. Some of the genes near AR-occupied regions appear to be regulated by the AR *in vivo* as evidenced by their expression levels in prostate cancer tumors of various stages. Several AR target genes discovered in the present study, for example PRKCD and PYCR1, may open avenues in PCa research and aid the development of new approaches for disease management.

## Background

Prostate Cancer (PCa) is the most commonly diagnosed non-cutaneous cancer and the second leading cause of cancer-related mortality in men [1]. Prostate development and carcinogenesis are highly androgen dependent [2,3]. By regulating cell proliferation, differentiation and apoptosis the androgen receptor (AR) plays a pivotal role in PCa progression, as well as in normal prostate development [2-4]. AR-mediated PCa growth is initially hormone-dependent, and men failing surgical and radiation therapy are therefore subjected to androgen ablation therapy [5]. Androgen ablation in these cases almost always leads to tumor regression, but this is inevitably followed by recurrence of PCa due to the development of castrate-resistant and often metastatic disease.

Although most recurrent PCa tumors are castrate-resistant, AR expression and function are maintained in advanced disease [6,7] and the growth of ablation-resistant PCa cells remains AR dependent as exemplified by the following three lines of evidence. Disruption of the AR by a specific antibody or ribozyme inhibited proliferation in ablation-resistant PCa cells in the absence of androgens [8]. Increased AR expression was necessary and sufficient to convert androgen-sensitive PCa to an ablation-resistant state [9]. Finally, specific expression in mouse prostate epithelial cells of an AR transgene containing a gain-of-function mutation (with increased basal activity and response to coregulators), resulted in PCa development in 100% of the animals [10] proving that aberrant AR signaling was sufficient to cause PCa and that under certain conditions the AR acts as an oncogene.

As AR is a transcription factor, its oncogenic functions are likely mediated through specific target genes. Prostate specific antigen (PSA), the best studied AR target gene, is thought to contribute to PCa progression through its protease activity [11] and its ability to induce epithelial-mesenchymal transition and cell migration [12]. Other AR target genes implicated in PCa progression are FGF8 [13], Cdk1 and Cdk2 [14], as well as PMEPA1 [15] and TMPRSS2 [16]. Interestingly, the AR response mechanism of TMPRSS2 drives oncogenic Ets family members in many castrate resistant tumors due to TMPRSS2:Ets chromosomal translocations [17,18]. However, additional, yet unidentified target genes most likely contribute to the tumorigenic activity of the AR in PCa. The present study was undertaken to identify such genes based on their physical interaction with the AR. C4-2B human PCa cells, a model for castrate-resistant disease, were subjected to a procedure called Chromatin Immunoprecipitation (ChIP) Display (CD) [19] and 19 novel regions occupied by the AR were discovered. The expression patterns of genes within the AR-occupied loci, along with functions attributed to these genes, render some of them potential PCa therapeutic targets.

## Results

### ChIP Display of AR targets in C4-2B cells: an example

**Figure 1:**
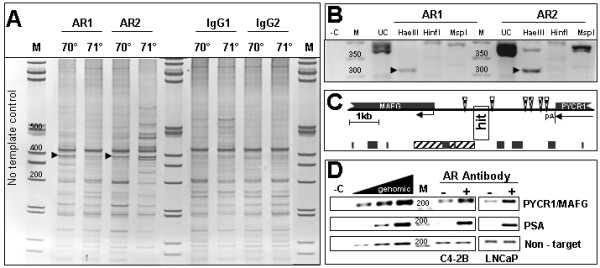
**ChIP Display (CD) demonstrates a putative AR target**. **A) CD Gel**. C4-2B cells were treated with 10 nM DHT for 4 hours to enhance AR association with target loci. Two independent AR ChIPs, and IgG control ChIPs were subjected to the CD procedure as described in Methods. In the example shown here, PCRs were performed with the 'AC' and the 'TG' PCR primers (see Methods and Additional file 1) with the annealing temperature set at either 70°C or 71°C as indicated. Amplified products were resolved using 8% PAGE and visualized by EtBr staining. The arrowheads point at bands amplified more prominently in the AR compared to the Control (IgG) lanes. *M*, marker DNA; numbers above bands indicate size in bps. **B) Re-amplification and digestion**. The two bands indicated in panel A by arrowheads were excised, purified and re-amplified with the same 'AC' and 'TG' primers used for CD. The products were subjected to secondary digestion with the indicated enzymes, followed by agarose gel electrophoresis. Arrowheads point at similar *Hae*III sub-fragments obtained from the two AR ChIPs. – *C*, no template control, *UC*, uncut, *M*, marker DNA. **C) Mapping of AR target**. The *Hae*III subfragments from B were excised, purified and sequenced. By blasting against the human genome using Ensembl [65], both sequences mapped to chromosome 17q25.3, ~1.5-kb upstream of the MAFG gene and ~2.5-kb downstream of the PYCR1 gene as shown in the diagram. The two genes are transcribed in the same direction as indicated by the horizontal arrows. *pA*, polyadenylation signal. The AR binding region discovered through CD (''*hit*'') abuts a CpG island (*bottom, striped rectangle*), but does not overlap with any repetitive elements (*bottom, black rectangles*). Several AREs (*checkerboard triangles*) were identified in this region using Consite [66]. **D) Validation of target by conventional ChIP analysis**. AR occupancy at the PYCR1/MAFG locus was tested by conventional ChIP assay. The PSA enhancer serves as positive control. A non-target locus serves as the negative control. Genomic DNA was used to demonstrate that the ChIP amplification was performed within a dynamic range. – *C*, no template control. *M*, marker DNA.

To identify AR targets in PCa, we employed ChIP Display, a newly developed method for the identification of regions occupied by transcription factors in living cells [19]. C4-2B human PCa cells were stimulated by androgens for 4 hours and ChIP was performed with either AR or IgG control antibodies. The purified DNA was digested with *Ava*II in order to standardize all DNA fragments representing each AR-occupied region to one size. The *Ava*II fragments were amplified using ligation-mediated PCR with each of 36 possible nested primer combinations [19]. The use of nested primers reduces ChIP noise because all the fragments representing a given locus are amplified with the corresponding primer combination, while non-specifically precipitated fragments are scattered, i.e. amplified with other primer combinations [19]. The PCR products are subsequently resolved by polyacrylamide gel electrophoresis (PAGE). Figure 1 describes an example of the procedure leading to the identification of one novel target, and Table 1 summarizes all the AR targets identified in this study.

**Table 1:** AR targets identified in this study

**CD Primers**^**1**^	**Band **^**2**^	***Ava*II – *Ava*II **^**3**^	**Nearby Genes **^**4**^	**Position of CD Hit Relative to gene**	**ChIP validation**^**5**^	
					
					**C4-2B**	**LNCaP**
AT, TA	1p35.2	30,152,547 – 30,152,728	Nearest gene is 626-kb away		4	2

AA, TC	1q25.2	178,433,288 – 178,433,456	***QSCN6 ***[63, 67]	***exon 13 ***	3	1
			LHX4 [68]	32.7-kb 5'		
			CEP350	84.2-kb3'		
			ACBD6	90.6-kb 3'		

AT, AT	2q37.3	241,348,804 – 241,348,990	***KIF1A***	***intron 23/exon 24***	3	1
			AQP12	62.4-kb 3'		

TT, TT	3p21.1	53,169,093 – 53,169,401	***PRKCD ***[51, 53]	***0.8-kb 5' ***	4	nd

AT, AC	4p16.1	6,644,411 – 6,644,619	***MAN2B2***	***intron 5***	3	3
			MRFAP1	48.7-kb 5'		

AA, TC	7q11.23	72,483,118 – 72,483,317	***FZD9 ***[69]	***2.7-kb 5'***	4	nd
			BAZ1B	10k-b 3'		

AT, AG	7q11.23	72,922,165 – 72,922,474	***WBSCR28***	***4-kb 3'***	4	2
			WBSCR27	27.4-kb 5'		
			CLDN4 [70]	37.2-kb 3'		

AA, AG	8q24.3	143,094,298 – 143,094,518	Nearest gene is 197kb away [64]		3	2

AC, TC	10p12.1	24,584,349 – 24,584,579	***KIAA1217***	***intron 2 ***	4	3

AT, AG	10q26.13	126,072,189 – 126,072,473	***OAT***	***3.4-kb 3'***	2	0
			LHPP	67.9-kb 5'		

AC, TC	11p15.4	1,017,234 – 1,017,529	***MUC6 ***[58]	***10-kb 5'***	3	3
			AP2A2	15-kb 3'		

AT, AT	11q12.3	62,532,814 – 62,532,977	***SLC22A8***	***intron 2***	5	2
			SLC22A6	23.9-kb 5'		
			CHRM1	87.3-kb 5'		

AT, AT	11q25	134,102,859 – 134,103,167	Nearest gene is 315-kb away		2	3

AT, AT	14q31.3	86,510,091 – 86,510,360	Nearest gene is 959-kb away		3	2

AT, AC	17p13.2	3,446,846 – 3,447,080	***TRPV1***	***exon 1/intron 1 ***	3	1
			CARKL	11.4-kb 3'		
			TRPV3 [60, 61]	39-kb 5'		

AC, TG	17q25.3	77,480,155 – 77,480,527	***MAFG***	***1.5-kb 5'***	4	2
			PYCR1 [36, 50]	2.5-kb 3'		
			SIRT7 [71, 72]	12-kb 5'		

AA, AA	22q11.23	22,655,127 – 22,655,462	***GSTT2 ***[62]	***exon 4/intron 4***	3	2
			DDT	3.1-kb 5'		

AG, AG	22q13.1	38,101,611 – 38,101,989	***SYNGR1 ***	***intron 2 ***	3	2
			MAP3K7IP1	25-kb 5'		

AC, TC	22q13.3	48,707,684 – 48,707,956	***CRELD2***	***1-kb 3'***	3	1
			ALG12	10-kb 5'		

^1^CD primers are defined based on two variable nucleotides as described in Methods.

^2^Cytogenetic band containing the CD hit.

^3^Absolute positions of the *Ava*II sites flanking the fragment displayed by PAGE.

^4^The nearest Refseq gene annotated in Ensembl [65] is shown in ***bolded and italicized ***text. Published papers suggesting relevance to cancer are referenced near the respective gene name.

^5^Number of independent conventional ChIP assays in which AR occupancy was confirmed. *nd*, not determined.

The example shown in Figure 1A entails the amplification and PAGE of two independent AR-ChIPs and two mock ChIPs using one of the 36 primer combinations – the 'AC' and the 'TG' primer (see Methods and Additional file 1). The arrowheads in Figure 1A point to bands more prominently amplified in the AR ChIPs as compared to the IgG ChIPs. These bands, representing a putative AR binding region, were excised, reamplified and further characterized by secondary restriction digests and agarose gel electrophoresis (Figure 1B). The major *Hae*III digestion product from each of the two ChIPs was sequenced and mapped to human chromosome 17q25.3, 1.5-kb upstream of the MAFG gene and 2.5-kb 3' of the PYCR1 gene (Figure 1C). The *Ava*II fragment displayed in the original PAGE (Figure 1A), depicted in Figure 1C as "hit", does not contain repetitive sequences and is located between two canonical Androgen Receptor Elements (AREs) (Figure 1C). A 2.4-kb CpG island is present adjacent to the hit (Figure 1C).

To validate AR occupancy at the region described above, we performed conventional ChIP assays with locus-specific primers (see Additional file 1). Four independent experiments with C4-2B cells showed that the PYCR1/MAFG locus was enriched in AR ChIPs as compared to paired IgG control ChIPs (Table 1, and see a representative result in Figure 1D).

### ChIP Display discloses 19 novel AR binding sites in PCa cells

The CD procedure, exemplified above for the 'AC' and 'TG' primer pair, was performed using all 36 possible primer combinations [19], resulting in the identification of 19 novel AR-occupied regions in C4-2B cells (Table 1). AR occupancy at the novel AR binding regions was confirmed in independent conventional ChIP assays of C4-2B cells (Table 1). Whereas only four of the 19 AR binding regions were up to 10-kb 5' of the nearest gene (indicated by bolded and italicized text in Table 1), many of the binding regions were either within the body of annotated genes (8 of the 19 regions) or up to 4-kb 3' of the nearest gene (3 of the 19 regions), indicating that AR-bound regions are not preferentially found within so-called 5'-flanking gene regulatory sequences. Four of the 19 CD hits were mapped to regions more than 197-kb away from any annotated gene (Table 1).

An important enigma in prostate cancer research is the molecular nature of the transition from androgen-dependent to castrate-resistant disease. In this context, the C4-2B cell line serves as a model of the latter, whereas its parent cell line, LNCaP, serves as a model of the former [20]. We speculated that many of the AR-occupied regions in C4-2B cells could become targets for this transcription factor only during the transition from androgen dependence to castrate-resistance and would therefore not be occupied by the AR in LNCaP cells. However, results of ChIP analysis in LNCaP cells were inconsistent with this notion, as 16 of 17 regions that we tested, which were occupied by the AR in C4-2B cells, were also occupied in LNCaP cells at least in one conventional ChIP assay (Table 1). Be that as it may, several of the AR-occupied regions are located near genes that have been linked to prostate or other cancers (see Discussion below and references in Table 1).

### AR occupied regions are associated with DHT-stimulated and DHT-repressed genes in C4-2B cells

**Figure 2:**
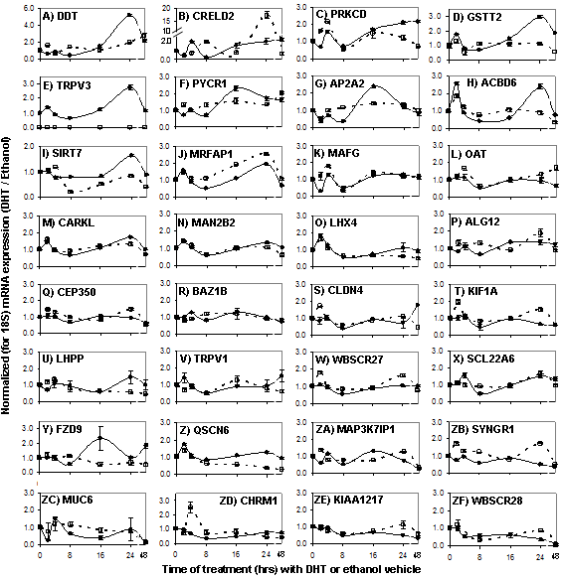
**Gene expression analysis**. C4-2B (solid lines) and LNCaP cells (broken lines) were maintained in 5% CSS-containing medium for three days, and then re-fed (time 0) with the same medium supplemented with either 10 nM DHT or ethanol vehicle. RNA was extracted at the indicated time points during the time course and expression of the specified genes was measured by RT-qPCR. Expression levels relative to 18S rRNA (which itself stayed stable throughout the time course) are shown with the 0 time values defined as 1 for each cell line. Representative data is shown from one of two independent experiments with n = 3, except for panels 2L, O, U, Y and ZC, where the C4-2B data is derived from 6 measurements (see Additional file 3 for the complete set of raw data). Error bars are SEM. Genes are roughly ordered based on the DHT-responsiveness in C4-2B cells, with stimulated genes first (panels A-J) to repressed genes last (panels ZC-ZF). TRVP3 mRNA was barely detectable in LNCaP cells.

One of the goals of this study was to identify primary AR-responsive target genes in PCa cells. Of the 19 AR-occupied regions, 15 were within 10-kb of Refseq-annotated genes. We initially measured the androgen responsiveness of genes nearest to each of these 15 AR-occupied regions (gene names bolded and italicized in Table 1). C4-2B cells were depleted from steroids, and treated with DHT or vehicle for 0, 2, 4, 8, 16, 24 or 48 hours. Gene expression was assessed by RT-qPCR. Of the 15 genes nearest AR occupied regions, expression of all but SLC22A8 was detectable, and only 6 of the remaining 14 genes responded to DHT treatment in a consistent manner. CRELD2, PRKCD and GSTT2 were stimulated (Figure 2B, C, D, solid lines), whereas MUC6, KIAA1217, and WBSCR28 were repressed (Figure 2ZC, ZE, ZF, solid lines). Because the remaining 8 of 15 genes nearest the AR occupied regions did not respond to DHT, we tested the expression of 19 additional nearby genes, up to 100-kb away from AR occupied regions. Of these 19 genes, the expression of all but AQP12 was detectable, but only eight responded to DHT treatment. DDT, TRPV3, PYCR1, AP2A2, ACBD6, SIRT7 and MRFAP1 were stimulated (Figure 2A and 2E–J, solid lines), and CHRM1 was repressed (Figure 2ZD). Altogether, of 32 genes within 100-kb from AR-occupied regions, many of which have been implicated in cancer progression (see references next to gene names in Table 1), ten were stimulated and four were repressed in DHT-treated C4-2B cells. More detailed investigation of the repressed genes is described elsewhere [21]. Notably, there were four loci in bands 2q37.3, 7q11.23, 10q26.13 and 22q13.1, where no nearby genes responded to DHT despite AR occupancy (Table 1 and Figure 2).

### AR-dependent, DHT-independent regulation of OAT and MRFAP1

**Figure 3:**
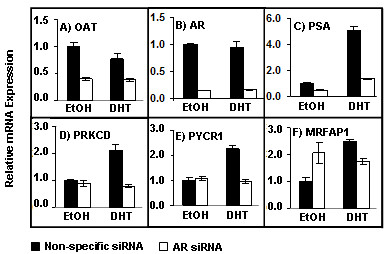
**Effects of AR siRNA-knockdown on gene expression**. C4-2B cells were treated with AR siRNA (white bars) or a non-specific siRNA (black bars), followed by administration of either DHT (10 nM) or Ethanol vehicle for 16 hours. Expression levels of the indicated genes were analyzed in triplicate by RT-qPCR and corrected for 18S rRNA levels. Values measured with the non-specific siRNA and ethanol were defined as 1. Results (Mean ± SD) are representative of three independent experiments.

Genes near AR-occupied regions that did not respond to DHT could still be regulated by the AR in a ligand-independent manner. To address this possibility, we treated C4-2B cells with AR siRNA duplexes [22] and assessed the effects on gene expression in the absence (and presence – as control) of DHT. Of eight genes near the four AR-occupied regions that were not associated with DHT-responsiveness in C4-2B cells, we found one, OAT, which was repressed in three of three siRNA experiments (Figure 3A), suggesting that it is indeed stimulated by the AR in the absence of ligand, despite its DHT non-responsiveness (Figure 2L). The other seven genes, KIF1A, AQP12, FZD9, BAZ1B, LHPP, SYNGR1 and MAP3K7IP1 did not respond to the siRNA treatment (data not shown), suggesting that AR occupancy at these loci may be without functional consequences in cultured C4-2B cells.

As controls for the AR siRNA experiments we also measured expression of AR itself and the DHT-stimulated genes PSA [23], PRKCD, PYCR1 and MRFAP1 (Figure 2C, F, J). As expected, the AR knockdown (Figure 3B) was associated with loss of DHT-stimulation (Figure 3C, D, and 3E). Interestingly, however, one of these controls, MRFAP1, displayed an unexpected phenotype. In addition to the DHT-stimulation, it was reproducibly stimulated in cells treated with AR siRNA (Figure 3F). Taken together, our data suggest that unliganded AR supports basal OAT expression (Figure 3A) without further stimulation by DHT (Figure 2L), while basal MRFAP1 expression is suppressed by unliganded AR (Figure 3F), yet stimulated by DHT (Figure 2J).

### Differential regulation of genes near AR-occupied regions in LNCaP versus C4-2B cells

Although most of the regions occupied by the AR in C4-2B cells (a model of castrate-resistant PCa) were also occupied in LNCaP cells (a model of androgen-dependent PCa) (Table 1), we suspected that the functional consequences of AR occupancy at these loci might differ between the two cell lines. We therefore complemented the DHT time course studies in C4-2B cells (Figure 2, solid lines) with parallel expression analysis of the same genes in LNCaP cells under a similar experimental protocol (Figure 2, dashed lines). Both similarities and differences between the two cell lines were observed. The three genes most strongly stimulated by DHT in C4-2B cells – DDT, CRELD2 and PRKCD – were also stimulated in LNCaP cells (Figure 2A, B, C), although the stimulation of DDT and PRKCD was more modest in LNCaP cells. Genes that were more moderately stimulated by DHT in C4-2B cells were slightly (PYCR1, Figure 2F) or not at all stimulated in LNCaP cells (GSTT2, AP2A2, ACBD6, and SIRT7; Figure 2, panels D, G, H, I). Repressed genes displayed a mirror image. The four genes most strongly repressed in C4-2B cells – MUC6, CHRM1, KIAA1217, and WBSCR28 – were also repressed in LNCaP cells, but repression generally occurred faster in the C4-2B cells (Figure 2ZC, ZD, ZE, ZF). Subtle responses to DHT were less consistent between C4-2B and LNCaP cells, although three genes, CARKL, MAN2B2, and LHX4, displayed remarkably similar expression patterns (Figure 2, panels M, N, O).

**Figure 4:**
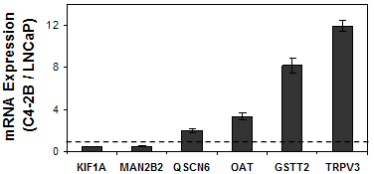
**Gene expression in C4-2B versus LNCaP cells**. RNA was extracted from C4-2B and LNCaP cultures that were maintained for two days in CSS-supplemented medium. Gene expression was analyzed side-by-side by RT-qPCR and corrected for 18S rRNA. Bars represent the comparative ratio between the expression in C4-2B and LNCaP cells, where the expression level in LNCaP cells is defined as 1. Included in this Figure are only genes for which the expression levels were significantly different between the two cell lines in two independent experiments (n = 3; Mean ± SD). TRPV3 mRNA was detected in LNCaP cells in only one of the three measurements, and this value was used as the upper limit for TRPV3 expression in these cells. See additional file 4 for details.

An emerging concept in PCa research is that ligand-independent AR-mediated gene expression contributes to the acquisition of a castrate-resistant growth state. If the derivation of C4-2B from LNCaP cells [20] were associated with such a mechanism, then one could expect expression of some genes near AR-occupied regions to be higher in hormone-deprived C4-2B as compared to hormone-deprived LNCaP cells. We therefore compared expression of the 32 genes near the AR-occupied regions between the two cell lines, and found four that were expressed in C4-2B cells at levels between 2 and 12-fold higher than in LNCaP cells (Figure 4). Not surprisingly, one of these genes was OAT, which was repressed after AR knockdown in C4-2B cells (Figure 3A). The other three were QSCN6, GSTT2, and TRPV3. Interestingly, two genes, KIF1A and MAN2B2, were 2 fold less expressed in C4-2B than in LNCaP cells. Thus, differences in gene expression between LNCaP and C4-2B cells, both under androgen deprivation and after DHT stimulation, may be involved in mechanisms of progression from early to late stage disease.

### Clinical relevance of novel AR target genes

**Figure 5:**
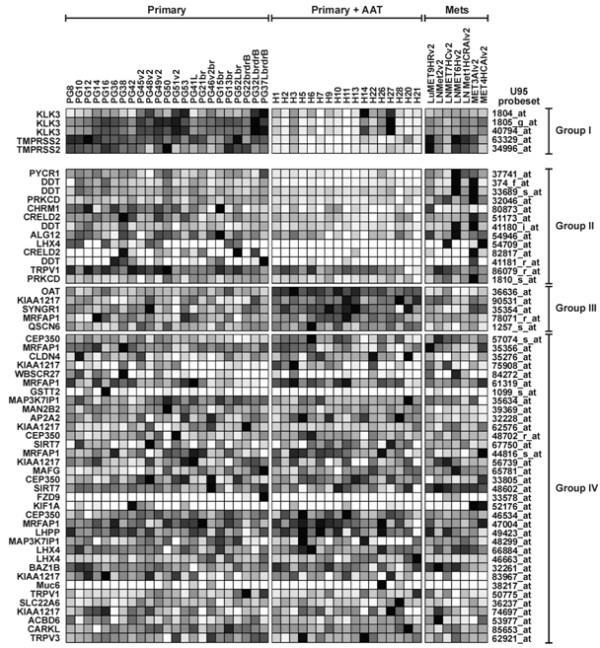
**Expression of CD-disclosed genes in PCa tumors**. RNA from 47 PCa tumors (columns) was analyzed using Affymetrix U95 A-E microarray sets (21) and results are mined for all probesets (rows) interrogating each of the 32 CD-disclosed genes (Table 1). Heat map shows relative expression for each of the indicated probesets, where darker shades represent higher mRNA levels. Tumors included 23 *primary *prostate cancers from patients not receiving therapy (*primary*), 17 primary prostate cancers following 3-month neoadjuvant androgen ablation therapy (*primary+AAT*), and 7 AR-positive metastatic lesions (*mets*). All Grade A probesets interrogating each gene are shown, except for probesets 59776_at (WBSCR28) and 36904_at (KIF1A), which did not detect significant expression in any sample. Samples are grouped and ranked as follows. *Group I *– probesets for the known AR-stimulated genes KLK3/PSA and TMPRSS2. *Group II *– probesets exhibiting statistically greater mean expression in untreated compared to AAT-treated primary PCa samples (*p *< 0.05), thereby representing putative AR-stimulated genes. *Group III *– probesets exhibiting statistically lower mean expression in untreated compared to AAT-treated PCa samples (p < 0.05), thereby representing putative AR-repressed genes. *Group IV *– probesets exhibiting no statistical difference between samples without or with AAT. Probesets in Groups II-IV are ranked by *p*-value in descending order.

To examine whether genes found in proximity to AR-occupied regions in our culture model are potentially regulated by the AR during PCa progression, we mined our microarray database of gene expression profiles in PCa tumors [24]. Figure 5 illustrates expression of the *in vitro *CD-disclosed genes in 23 untreated primary PCa tumors, 17 primary tumors after 3 months of androgen ablation therapy and 7 AR-positive metastatic tumors. Expression of several of these genes was consistent with *in vivo *regulation by the AR. As shown in Figure 5, Group II, the mRNAs for PYCR1, DDT, PRKCD, and CRELD2, which were DHT-stimulated *in vitro *(Figure 2), were decreased in the androgen-ablated as compared to the primary untreated tumors. Furthermore, when compared to the androgen-ablated tumors, the expression of these four genes was elevated in the metastatic tumors (Figure 5), presumably due to reactivation of the AR [5,25]. The similarity between the expression profiles of the CD-disclosed targets PYCR1, DDT, PRKCD, and CRELD2 in the clinical samples and those of the established AR target genes PSA/KLK3 [11] and TMPRSS2 [16] (Figure 5, Group I) suggests that the four genes discovered in our *in vitro *study are indeed AR targets *in vivo*. Interestingly, expression of ALG12 and CHRM1, which were unresponsive or even repressed by DHT *in vitro*, were decreased in androgen-ablated as compared to untreated primary tumors (Figure 5, group II), suggesting positive regulation by the AR *in vivo*, possibly via mechanisms not operative in our cell culture system. Five probesets displayed a profile indicative of AR-mediated repression *in vivo *(Figure 5, Group III). Of the corresponding five genes, KIAA1217 was strongly inhibited, while QSCN6 and SYNGR1 were only slightly inhibited by DHT *in vitro *(Figure 2Z and 2ZB). Notably, the evidence for KIAA1217 repression *in vivo *was provided by only one probeset (located at the 3'UTR, close to the region targeted by our RT-qPCR primers) and not by five other KIAA1217 probesets present on the array (Figure 5, Group IV). The remaining two genes in Group III (Figure 5), MRFAP1 and OAT, appear to be negatively regulated by the AR *in vivo*, although they were stimulated or non-responsive to DHT *in vitro *(Figures 2J and 2L). Interestingly, both these genes were regulated by unliganded AR *in vitro *(Figure 3). Thus, the expression profiles in the PCa biopsies suggest AR-mediated regulation of CD-disclosed AR target genes *in vivo*, although the nature of the *in vivo *response is not always consistent with that seen *in vitro*.

## Discussion

### AR occupancy is not biased towards 5' promoter-proximal regions

The classical view of gene regulation places 5'-flanking sequences at the center stage. Consistent with this view, functional AREs have been mapped within 0.5-kb upstream of the AR-responsive genes probasin, KLK2 and KLK3 (PSA) [26-28]. While four of the 19 AR-occupied regions disclosed in our study were located within 10-kb upstream of annotated transcription start sites, many more were found within gene bodies (8/19) or within the 4-kb sequences downstream from the 3' ends of annotated genes. Our findings are consistent with several recent genome-wide location analyses of other transcription factors. For example, only 4% of estrogen receptor (ER) binding sites were mapped to 1-kb promoter-proximal regions by ChIP-chip analysis [29]. Similarly, genome-wide location analysis indicates that p53 has no preference for binding to 5' promoter-proximal regions [30]. Thus, accumulating evidence suggest that promoter-proximal regions constitute only a small fraction of mammalian gene regulatory sequences.

Many of the AR-occupied regions identified in the present study, which cannot be designated classical 5' promoter regions, were still close to annotated genes that they could potentially regulate. There is no consensus as to how far a transcription factor-binding region should be in order to be considered a putative cis-acting regulatory domain for a given gene. Values 1-kb, and up to 100-kb from the transcription start site have been used by various investigators [29,30], but experimental evidence in support of any value is scarce. Systematic analyses of transcription factor binding regions across the genome have become feasible only recently. Such studies, including the present one, illustrate the need for mutagenesis of transcription factors binding regions that are distant from annotated genes in order to identify functionally relevant regions. In particular, it would be interesting to decipher the role of binding regions located hundreds of kbs away from the nearest annotated gene. Such regions may still regulate distant annotated genes on the same [31] or even other chromosomes [32], or they may regulate nearby unannotated transcripts [33].

### AR location analysis discloses ligand-independent, AR-dependent gene regulation

Comprehensive gene expression analysis is frequently employed for the discovery of target genes for transcription factors, including the AR [24,34-36]. Such expression analyses cannot differentiate between direct and indirect targets and they do not provide information on the location of regulatory elements. Another, frequently under-appreciated limitation of expression studies is that they only disclose target genes that respond to the transcription factor of interest under the specific experimental conditions utilized by the investigator. In contrast, ChIP Display and other approaches for location analysis (see below) rely on physical interaction, not gene expression. These approaches allow the discovery of target genes that expression studies would potentially miss. For example, in the present study, we discovered OAT as an AR-regulated gene, although it did not respond to DHT. AR-target genes could also be missed in expression-based studies because of the limited sensitivity and specificity of microarray hybridization as compared to RT-qPCR. Experimental approaches for location analysis are constantly improving and include many that are far more comprehensive than ChIP Display, for example ChIP-chip [37-39], SABE [40], STAGE [41], ChIP-PET [30], GMAT [42], SACO [43] and DamID [44]. However, the present study demonstrates that important information can be obtained with ChIP Display, a relatively inexpensive sampling method that can be performed in any molecular biology laboratory. Of course, each of the expression and the location approaches for target identification should ideally be complemented appropriately. In the present study, we showed that many (but not all) of the genes near AR-occupied regions are DHT-responsive. For OAT, which was disclosed here by ChIP Display and could not have been disclosed by comprehensive analysis of gene expression in response to androgen treatment, we used siRNA knockdown to demonstrate the ligand-independent regulation by the AR. Furthermore, of the genes near AR-occupied regions that responded to neither DHT nor AR siRNA in our study, some may be AR-regulated under specific, possibly transient physiological or pathological conditions not modeled by the experimental systems we employed. This is particularly important in the context of castrate-resistant PCa, where AR activation can occur through various signaling pathways, including Her2, AKT, and MAPK [25].

### Differential basal gene expression in C4-2B versus LNCaP cells

The AR plays critical roles during all stages of PCa progression [5,9,45]. It is not clear, however, whether AR regulates different sets of genes before and after ablation therapy. In our study, AR occupancy at most of the regions disclosed by CD was similar in LNCaP and C4-2B cells, models of early and late stage PCa, respectively. Our data is therefore consistent with the idea that the AR continues to regulate the same genes before and after ablation therapy, but that the nature of this regulation alters during disease progression. For many genes near AR-occupied regions, ligand-bound AR had the same qualitative effects in the two cell lines, except they were stronger in the C4-2B as compared to the LNCaP model (e.g., DDT, PRKCD, GSTT2, PYCR1; Figure 2). Some other genes near AR-occupied regions were found to express at higher basal levels in C4-2B as compared to LNCaP cells (e.g., OAT, GSTT2, TRPV3; Figure 4). The higher basal expression of these genes could be a direct result of ligand-independent activation by the AR due to, for example, cofactor expression [46,47] and/or chromatin reorganization [22]. Accumulation of mutations during the derivation of C4-2B from LNCaP cells could also contribute to differential basal and DHT-responsive expression, although the two cell lines are mostly isogenic as indicated by our microsatellite analysis (see Additional file 2).

### Evidence from PCa biopsies for in vivo AR-mediated regulation of genes disclosed by ChIP Display

In the present study, we included analysis of the ChIP Display-disclosed genes for their expression in PCa biopsies. This analysis provided evidence that AR regulates *in vivo *several of the ChIP Display-disclosed genes. This notion, however, remains tentative because the clinical material used for the microarray expression analysis is no longer available for confirmation by RT-PCR. To increase our confidence in the microarray data, we only considered results from probesets that are considered highly reliable (Affymetrix' grade A annotation). Results from the PCa biopsies were consistent with those from the *in vitro *gene expression analysis for the positively regulated genes PYCR1, DDT, PRKCD, and CRELD2. However, ALG12 and TRPV1, which appear to be stimulated by the AR *in vivo*, were not responsive to either DHT or AR siRNA *in vitro*. The *in vivo *and *in vitro *analyses were less well correlated for negatively regulated genes. Only one of six probesets interrogating KIAA1217 expression indicated repression *in vivo*, although this gene was strongly inhibited *in vitro*. CHRM1 was also strongly repressed by androgens *in vitro*, yet it was found to be stimulated *in vivo*. OAT, which appears to be downregulated by the AR *in vivo*, was stimulated *in vitro *in a ligand independent manner. It remains to be seen whether these inconsistencies result from differential requirements for AR-mediated gene stimulation/repression *in vitro *and *in vivo*, the presence of multiple splicing isoforms, or simply erroneous microarray expression scores. Be that as it may, further investigation of genes highlighted by our study, and especially genes for which results from the PCa biopsies are apparently inconsistent with the *in vitro *results, will have to start with validation of the regulation of such genes in PCa *in vivo*.

### Novel AR target genes: potential mechanisms contributing to PCa progression

PSA (KLK3) remains the most well studied AR target gene in the PCa literature to date. Since its approval in 1986, serum PSA is routinely used to aid the early diagnosis and prognosis of PCa in men. However, AR-driven PSA expression alone does not fully explain the role of AR in PCa development and progression. Although additional AR target genes have been recently discovered, e.g., FKBP5 [48] and TMPRSS2 [16], most remain elusive. Some of the AR target genes discovered in the present study, and more to be discovered in the future, may open new research avenues and help develop novel therapeutic approaches to manage PCa.

#### PYCR1

Pyrroline-5-carboxylate reductase 1 (PYCR1) catalyzes the NAD(P)H-dependent conversion of pyrroline-5-carboxylate (P5C) to proline. Stimulation of PYCR1 by the AR could contribute to PCa progression because P5C is pro-apoptotic [49] and proline is anti-apoptotic [50]. Indeed, a role for PYCR1 in PCa was suggested by a 4-fold increased expression in human prostate tumors compared to adjacent normal tissue [36]. In the present study we demonstrate AR occupancy at the PYCR1 locus in living PCa cells, the functionality of which is suggested by DHT-mediated stimulation of gene expression. Consistent with these *in vitro *data, we also demonstrate decreased PYCR1 expression in PCa biopsies from men undergoing androgen ablation therapy as compared to untreated controls. The highest PYCR1 expression in our PCa samples was found in biopsies from metastatic tumors, possibly as a result of atypical AR activation. Of the AR targets discovered in the present study, PYCR1 is a strong candidate for mediating the oncogenic action of AR signaling in PCa.

#### OAT

Interestingly, another AR target gene discovered in this study also participates in proline metabolism. Ornithine aminotransferase (OAT) converts ornithine to glutamate γ-semialdehyde, which spontaneously cyclizes to form pyrroline-5-carboxylate (P5C), a proline precursor and the substrate for PYCR1. The functional evidence for AR-mediated regulation of OAT is weaker than that for PYCR1. While OAT mRNA was not significantly altered in response to DHT, it was repressed after siRNA-mediated knockdown of the AR, and also displayed a 3.3-fold higher basal expression in C4-2B as compared to LNCaP cells. Interestingly, OAT's expression pattern in our tumor samples does not suggest AR-mediated stimulation, but rather repression, possibly reflecting interactions of AR signaling with input from other cell types or components of the extracellular matrix, which only occur *in vivo*.

#### PRKCD

In this study, we mapped AR occupancy to a region 0.8-kb upstream of the gene encoding Protein Kinase C delta (PRKCD), which has received much attention in the PCa literature. PRKCD mRNA levels were higher in DHT-treated as compared to untreated PCa cell cultures and were reduced in PCa biopsies from patients undergoing androgen ablation therapy as compared to those from untreated patients. The observed high PRKCD mRNA levels in PCa metastases, which may reflect ligand-independent AR activation, possibly play a role in late stage disease because PRKCD is implicated in growth, migration and invasion of cancer cells, including PCa [51,52]. PRKCD has also been implicated in the control of cell survival, although most studies suggest it is in fact pro-apoptotic [53-55]. Future studies will have to address how PRKCD's pro-apoptotic activity is overcome in advanced PCa cells.

#### CRELD2 and DDT

The gene expression data form both the cell culture models and the clinical tumor samples suggest androgen-mediated stimulation of CRELD2 and DDT. Although a role for CRELD2 in carcinogenesis remains to be investigated, this Cysteine-rich with EGF-like Domains 2 (CRELD2) protein has been shown to interact with neuronal acetylcholine receptors [56]. Likewise, no role in tumor progression has been assigned yet to DDT, a protein with homology to macrophage migration inhibitory factor (MIF) [57].

#### MUC6

Among the few genes near AR-occupied regions that were repressed by DHT was MUC6. Mucin 6 is a secreted glycoprotein that forms a protective gel layer around the producing cell [58]. Other mucins are aberrantly expressed in cancer [58] and MUC2 was ascribed a tumor suppression function [59]. Conceivably, AR-mediated MUC6 repression can contribute to PCa progression. Notably, however, MUC6 mRNA was neither increased in the androgen-ablated compared to the untreated tumors, nor was it absent in the metastatic samples. Although our *in vivo *data does not support a role for MUC6 in PCa progression, MUC6 could still play a transient role during a short period of time not captured by our clinical samples.

#### TRPV3 and GSTT2

Like the androgen-repressed MUC6, evidence for roles for the androgen-stimulated TRPV3 and GSTT2 genes in PCa progression is suggested only from our *in vitro *data. These two genes are not only androgen stimulated but are also expressed in C4-2B cells more strongly than in LNCaP cells. TRPV3 is a member of the transient receptor potential (TRP) family of thermosensory ion channel genes. Another member of this family, TRPV6, potentiates calcium-dependent cell proliferation [60], and its expression has been linked to human PCa progression [61]. Glutathione S-transferase theta 2 (GSTT2) belongs to a family of detoxification enzymes, overexpression of which is thought to provide cells with protection against oxidative stress and various drugs [62].

#### AR occupancy at PCa-linked loci

Two of the AR-occupied regions disclosed by ChIP Display were near loci previously linked to PCa: (i) the hereditary prostate cancer 1 (HPC1) locus, which has been mapped to 1q24-25 [63]; and (ii) the 8q24 locus, recently linked to PCa through admixture mapping in African American men [64]. Although fine mapping of specific genetic elements has not been achieved yet for either of these loci, their contribution to PCa progression in a mechanistic sense may be related to the observed AR occupancy.

## Conclusion

We have identified 19 novel AR-occupied regions in PCa cells, many of which are associated with genes that are regulated by the AR in either a ligand-dependent or ligand-independent manner. Furthermore, some of the newly identified AR target genes are differentially regulated in cell models for, and/or biopsies from, different stages of PCa progression. These genes provide opportunities for future research to better understand the role of the AR in PCa and eventually improve patient care, especially in the context of castrate-resistant disease.

## Methods

### Cell culture and materials

Human C4-2B cells, a model for castrate-resistant PCa, were obtained from ViroMed Laboratories Inc. (Minnetonka, MN) and their parental, androgen-dependent LNCaP cells, were obtained from ATCC. (Manassas, VA). The close relationship between the two cell lines was confirmed by microsatellite analysis at ten loci (see Additional file 2). Both C4-2B and LNCaP cells were maintained in RPMI-1640 medium (Invitrogen, Carlsbad, CA) supplemented with 5% fetal bovine serum (FBS; Invitrogen). Dihydrotestosterone (DHT; Sigma Chemical Co., St. Louis, MI) was administered in phenol red-free RPMI-1640 supplemented with 5% charcoal-stripped FBS (CSS; Gemini, West Sacremento, CA). An N-terminal AR antibody (N20) was purchased from Santa Cruz Biotechnology (Santa Cruz, CA).

### ChIP

ChIP was carried out essentially as described previously [22]. C4-2B and LNCaP cells were cultured for 3 days in phenol red-free RPMI-1640 supplemented with 5% CSS, then treated for 4-hr with 10 nM DHT, followed by cross-linking with 1% formaldehyde for 10 minutes. After sonication, chromatin was immunoprecipitated overnight at 4°C with either anti-AR antibodies or isotype-matched IgG. AR occupancy was assessed by PCR with locus-specific primers (see Additional file 1) using material from several independent ChIPs. Serial dilutions of genomic DNA were amplified to ensure that PCR was performed within a dynamic range.

### ChIP Display (CD)

We have recently described the CD procedure in detail [19]. Briefly, DNA from AR ChIP and IgG control ChIP was dephosphorylated using shrimp alkaline phosphatase (NEB, Ipswich, MA) and digested with *Ava*II (NEB). The *Ava*II fragments were subjected to ligation-mediated PCR using each of 36 combinations of eight primers. Each primer had A or T at the +3 position of the *Ava*II site, and A,T,G, or C at the so-called +6 position, immediately internal to the *Ava*II site [19]. In the present paper, primers are named by the nucleotides occupying these two positions. For example, the PCR primer 'AC' is the one with A at the +3 and C at the +6 position. Each PCR reaction in the present study was performed in duplicate, with a 1°C difference in the annealing temperature (see Fig. 1A). The amplified material form 2–3 independent AR ChIPs and 2–3 controls was resolved by polyacrylamide gel electrophoresis (PAGE), and bands enriched in the AR ChIPs were excised and reamplified. They were then subjected to secondary digestion with *Hae*III, *Hinf*I and *Msp*I (NEB), and sub-fragments were isolated by agarose gel electrophoresis and sequenced. The sequences were mapped to the human genome using the SSAHA program on ENSEMBL [65].

### AR siRNA

C4-2B cells (1.5 × 10^5 ^cells/well in 6 well plates) were cultured for two days in phenol red-free RPMI-1640 supplemented with 5% CSS. The cells were then transfected using OligofectAMINE (Invitrogen) with 100 nM of either AR-specific or non-specific siRNA (see Additional file 1) as previously described [22]. After two days, cells were treated for 16 hours with 10 nM DHT or ethanol vehicle prior to analysis of gene expression.

### RT-PCR

Cells were grown in six-well plates and RNA was extracted using Biorad's total RNA mini kit according to manufacturer's protocols (Biorad, Hercules, CA). RNA quality was assessed spectrophotometrically and by agarose gel electrophoresis. High quality RNA (200–1000 ng) was reverse-transcribed with random hexamers using the Taqman reverse transcription reagents kit (Applied Biosystems, Foster City, CA). cDNAs of interest were amplified using gene specific-primers (see Additional file 1) and the iQSYBR Green supermix (Biorad). Amplification was performed in triplicate in a 96-well format and monitored in real time using the Opticon 2 DNA Engine (Biorad). Negative controls without RNA, without RT and without cDNA were always included to rule out contamination. Expression levels were determined using standard curves for each gene and corrected for 18S ribosomal RNA levels.

### Gene expression analysis in clinical PCa specimens

Expression of genes disclosed by ChIP Display was analyzed in prostate cancer samples using microarray data collected as part of our previous studies [24]. Briefly, clinical samples were from 40 primary prostate cancers obtained during radical prostatectomy and 7 AR-positive metastatic prostate cancer lesions. Twenty-three of the primary tumors were from patients receiving no therapy before surgery and the remaining 17 were from patients after 3 months of goserelin plus flutamide androgen-ablation therapy. All tissues were obtained during routine clinical management at the Memorial Sloan-Kettering Cancer Center, New York, NY, under protocols approved by the Institutional Review Board. RNA was extracted from manually-microdissected tissue consisting of 60–80% prostate cancer cell nuclei, and analyzed as previously described [24] using the Affymetrix U95 A-E array set. The results are displayed as a heat-map generated using 'Heatmap Builder' (Stanford University), with data routed to 50 equal gates for each probeset (row) using a linear grey scale gradient from white (lowest value) to black (highest value). Data for each gene was generated using only those high-fidelity probesets with a grade A annotation as defined by Affymetrix.

### Statistical analysis

We employed the unpaired *t*-test using GraphPad Instat version 3.0 for PC to compare mRNA levels for each gene between DHT-treated and vehicle-treated cells at each time point, and between the basal levels in LNCaP versus C4-2B cells. Unless otherwise stated, differences referred to in the text were assigned a *p *value of less than 0.05. The individual *p *values assigned to each of the comparisons are provided in additional files 3 and 4.

## Abbreviations

AR, androgen receptor; ChIP, chromatin immunoprecipitation; CD, ChIP Display; PCa, prostate cancer; PAGE, polyacrylamide gel electrophoresis; DHT, dihydrotestosterone; PSA, prostate specific antigen 1; CSS, charchoal stripped serum; FBS, fetal bovine serum; ARE, androgen responsive element; RT-qPCR, real time quantitative polymerase chain reaction; ER, estrogen receptor; SABE, serial analysis of binding elements; STAGE, sequence tag analysis of genomic enrichment; ChIP-PET, chromatin immunoprecipitation paired-end ditag sequencing; GMAT, genome mapping technique; SACO, serial analysis of chromatin occupancy; DamID, tethered Dam methyltransferase identification; P5C, pyrroline 5-carboxylate; DDT, D-dopachrome tautomerase; CRELD2, cysteine-rich with EGF-like Domains 2; PRKCD, protein kinase C delta; GSTT2, glutathione S-transferase theta 2; TRPV3, transient receptor potential (TRP) subfamily V member 3; PYCR1, pyrroline-5-carboxylate reductase 1; AP2A2, adaptor-related protein complex 2 alpha 2 subunit; ACBD6, Acyl-Coenzyme A binding domain containing 6; SIRT7, silent mating type information regulation 2 (sirtuin 7); MRFAP1, mof4 family associated protein 1; MAFG, v-maf musculoaponeurotic fibrosarcoma oncogene homolog G; OAT, ornithine amino transferase; CARKL, carbohydrate kinase like; MAN2B2, mannosidase 2 alpha B2; LHX4, LIM homeobox 4; ALG12, alpha 1,6 mannosyltransferase; CEP350, centrosomal protein 350 kDa; BAZ1B, bromodomain adjacent to zinc finger domain 1B; CLDN4, claudin-4; KIF1A, kinesin family member 1A; LHPP, phospholysine phosphohistidine inorganic pyrophosphate phosphatase; TRPV1, transient receptor potential (TRP) subfamily V member 1; WBSCR27, williams beuren syndrome chromosome region 27; SCL22A6, solute carrier family 22 (organic anion transporter); FZD9, frizzled homolog 9; QSCN6, quiescin Q6; MAP3K7IP1, mitogen-activated protein kinase kinase kinase 7 interacting protein 1; SYNGR1, synaptogyrin-1; MUC6, mucin 6; CHRM1, cholinergic receptor, muscarinic 1; WBSCR28, williams beuren syndrome chromosome region 28.

## Competing interests

The author(s) declare that they have no competing interests.

## Authors' contributions

UJ – Wrote the manuscript, contributed substantially to ChIP Display, gene expression analysis, and summary of the data.

JP – Contributed substantially to ChIP Display, gene expression analysis and data interpretation.

LJ – Generated the ChIP material for ChIP Display and participated in the experimental design.

AB, SP – Processed the ChIP material for use in ChIP Display and participated in the experimental design.

GB – Extracted and analyzed the gene expression data from the microarray experiment.

JPC, AA – Contributed to ChIP Display.

HIS, WLG, WDT – Designed and supervised the microarray studies on the clinical tumor samples.

GAC, BF – Co-directed this study, corrected the manuscript.

*All authors read this manuscript*.

## Supplementary Material

Additional file 1 Oligonucleotides used in our studies. This file lists sequences of the oligonucleotides used for ChIP display, conventional ChIP assays, qPCR and siRNA. Click here for file

Additional file 2 Microsatellite analysis in LNCaP and C4-2B. This file provides the results of microsalletile analysis of C4-2B cells versus the parental LNCaP cells. Click here for file

Additional file 3 Complete dataset and statistical analysis of DHT-responsive gene expression in C4-2B and LNCaP cells. This file provides the raw data, which is summarized in Figure 2. Click here for file

Additional file 4 Complete dataset and statistical analysis of basal gene expression in C4-2B versus LNCaP cells. This file provides the raw data, which is summarized in Figure 4. Click here for file
