# Supplementary material for: Identification of novel androgen receptor target genes in prostate cancer
Source: Mol Cancer. 2007 Jun 6;6:39. doi: 10.1186/1476-4598-6-39 (PMC1904239; doi:10.1186/1476-4598-6-39)
Supplement: Additional file 1 — Oligonucleotides used in our studies. This file lists sequences of the oligonucleotides used for ChIP display, conventional ChIP assays, qPCR and siRNA. [file 1476-4598-6-39-S1.doc]

**Additional File 1**

| **A) CD Oligonucleotides** | |
| --- | --- |
| Short linker oligo | 5’ -TTC GCG GCC GCA C- 3’ |
| Long A linker oligo | 5’ -G**A**C GTG CGG CCG CGA A- 3’ |
| Long T linker oligo | 5’ -G**T**C GTG CGG CCG CGA A- 3’ |
|  |  |
| "AA" PCR primer | 5’ -CGG CCG CAC G**A**C C**A**- 3’ |
| "AT" PCR primer | 5’ -CGG CCG CAC G**A**C C**T**- 3’ |
| "AG" PCR primer | 5’ -CGG CCG CAC G**A**C C**G**- 3’ |
| "AC" PCR primer | 5’ -CGG CCG CAC G**A**C C**C**- 3’ |
| "TA" PCR primer | 5’ -CGG CCG CAC G**T**C C**A**- 3’ |
| "TT" PCR primer | 5’ -CGG CCG CAC G**T**C C**T**- 3’ |
| "TG" PCR primer | 5’ -CGG CCG CAC G**T**C C**G**- 3’ |
| "TC" PCR primer | 5’ -CGG CCG CAC G**T**C C**C**- 3’ |

**B) ChIP Primers**

| **Chr.**   | **Forward Primer** | **Reverse Primer** |  | | --- | --- | --- | | 1q25.2 | 5' -CCT TCC GGA CAA TGA AGA AG- 3' | 5' -AAG CAA GCC ACT CAC CCT AC- 3' | | 1p35.2 | 5' -AGG GAC AAC ATC ACC AGG AG- 3' | 5' -AGC ACG GCT ACT GCA CCT AC- 3' | | 2q37.3 | 5' -GCT TCT CCA GCG TCC AGT AG- 3' | 5' -CTG ACG AGT GGT CAT CTT CC- 3' | | 3p21.1 | 5' -CCA GAG AAC AGC ATG CTC AA- 3' | 5' -TAC AAC CAG GCA GGA TGA CA- 3' | | 4p16.1 | 5' -GTG GAA ACT GTT GGG TGG AG- 3' | 5' -CTG GTC CCC ACT GAG TCT TC- 3' | | 7q11.23 | 5' -CTCAAGAGGATCGGGAGATG- 3' | 5' -TCCTGGGCTCAAGTGATTCT- 3' | | 7q11.23 | 5' -ACC AGA GGG GTC TGT GTG TC- 3' | 5' -AAG CGA GAA GCG CTA ATA GG- 3' | | 8q24.3 | 5' -GTG GAT GGA TGG CAA ATA GG- 3' | 5' -GCT CCT TTG TCA GGG ATC AG- 3' | | 10q12.1 | 5' -GTG GGC TCA GCA CTG GAC- 3' | 5' -CAG GGA AAC CCC AGA ATC AG- 3' | | 10q26.13 | 5' -CTC CCA AAC TCC TCC AAC TG- 3' | 5' -GGG TAG AAC ATC AGG GCA AC- 3' | | 11p15.4 | 5' -GTG ATG CCG TTG ATG ACA GT- 3' | 5' -ACG GGT AAC ACC ACC TTC AG- 3' | | 11q12.3 | 5' -CAT TAA GTC ATT GTA AGG CCT GTG- 3' | 5' -TCC AGG ATC AGG AAC TCA CC- 3' | | 11q25 | 5' -GTC CTA CCC TGG AGG GAC TG- 3' | 5' -GGA GGA GAG GAA TCG AGG TC- 3' | | 14q31.3 | 5' -CCA TCT GCT TAG ATG TTC ATG C- 3' | 5' -TGG GAT CTT TGA GGG GAT AAC- 3' | | 17p13.2 | 5' -GCA AAA ATG ATG GGA AAA GC- 3' | 5' -CTT GAA GGC GGT TGC TAC TC- 3' | | 17q25.3 | 5' -ACC CCA ACT CCT CCT CAC AG- 3' | 5' -CCA AGA AGA TTC TGG GGT GA- 3' | | 22q11.23 | 5' -GAT TGG CCA TCA GGG AGT AG- 3' | 5' -TGA GAT CAG CCA GTG TCA CC- 3' | | 22q13.1 | 5' -CAT GGG AGA TGC ACT CTT GA- 3' | 5' -GTT CAG TGG GTT GTC CTT GG- 3' | | 22q13.3 | 5' -ACC ACC CAC TCC TCA GTC AC- 3' | 5' -ACA GGG GCT CTT CCA ATA CC- 3' | | 11p11.2 (non-target) | 5' -CCG ACT TCC TCT CCT GAC TG- 3' | 5' -TCA GCT TGC TCC CCA TTT AT- 3' | | PSA enhancer | 5' -TGA AAA CAG ACC TAC TCT GGA- 3' | 5' -AGC AAA GAC AGC AAC ACC TT- 3' | |  |  |
| --- | --- | --- | --- | --- | --- | --- | --- | --- | --- | --- | --- | --- | --- | --- | --- | --- | --- | --- | --- | --- | --- | --- | --- | --- | --- | --- | --- | --- | --- | --- | --- | --- | --- | --- | --- | --- | --- | --- | --- | --- | --- | --- | --- | --- | --- | --- | --- | --- | --- | --- | --- | --- | --- | --- | --- | --- | --- | --- | --- | --- | --- | --- | --- | --- | --- | --- | --- | --- |
|  |  |  |
|  |  |  |
|  |  |  |
|  |  |  |
|  |  |  |
|  |  |  |
|  |  |  |

**C) RT-qPCR Primers**

| **Gene** | **Forward Primer** | **Reverse Primer** |
| --- | --- | --- |
| QSCN6 | 5' -ACC CTC AAC TTC CTC AAG- 3' | 5' -TCA TCA TCT CAG GCT TCC- 3' |
| LHX4 | 5' -CAG GCG GAC AGT TAA TGA ATG G- 3' | 5' -GGA CGA TAT GGA GGA TGG AGA C- 3' |
| CAP350 | 5' -AGA ATG GAG CCA AAA GAG CA- 3' | 5' -CAA GAA TGC CAC GAA TTT CA- 3' |
| ACBD6 | 5' -GGC CTG TGA TCG AGG ACA TA- 3' | 5' -TAA GCC TTG CCA GTT GTG TG- 3' |
| KIF1A | 5' -AAG GCC TCC TCC TAG ACA GC- 3' | 5' -CTG TGT TCT TCA GGG GCT CT- 3' |
| PRKCD | 5' -CCT GAC TAT ATC GCC CCT GA- 3' | 5' -GTC CTT GGA CTC CTT GGT GA- 3' |
| MAN2B2 | 5' -GGG TGT ACC CCA ACA TGA GT- 3' | 5' -CTG TGG AAT AGG GCA GGA AG- 3' |
| MRFAP1 | 5' -TGC TCA TCC AGA TCA AAA CG- 3' | 5' -CAA AAG GCT CTC TGG TTT CG- 3' |
| FZD9 | 5' -AGA CCA TCG TCA TCC TGA CC- 3' | 5' -CCG ATC TTG ACC ATG AGC TT- 3' |
| BAZ1B | 5' -AAA GCC TTC CAC CTG TTT TG- 3' | 5' -GCA AAC CAG CCA CCT CAT AA- 3' |
| WBSCR28 | 5' -AGT GAC CTG GAG GGT GTG TC- 3' | 5' -CTG GGT CGT GTG CTC AAA G- 3' |
| WBSCR27 | 5' -GTC TGA CCA CCA GGA CCA AC- 3' | 5' -AGA CAA TGC CGG AGA TGA AG- 3' |
| CLDN4 | 5' -TGC TTT GTT CTT CCC TGG AC- 3' | 5' -ACC ACC ACA CCC TGT CAC TT- 3' |
| KIAA1217 | 5' -CCA TGA GTG CCA AGA ACA GA- 3' | 5' -TTG ACT CTG CGG TGA GAA TG- 3' |
| OAT | 5' -TTC TGG GGT AGG ACG TTG TC- 3' | 5' -AGC TCT CGC ACT CCC ATT AG- 3' |
| LHPP | 5' -GAG GTT CTG CAC CAA CGA GT- 3' | 5' -CAC ACA GTT TGG GTT GGA TG- 3' |
| MUC6 | 5' -AAC ATC ATC ACC CAG CAG GT- 3' | 5' -TGG TGG GTG TTT TCC TGT CT- 3' |
| AP2A2 | 5' -TGA CGT CTG CAT CCA CAG AT- 3' | 5' -TGC TGG ACC TTC TTC GAC TT- 3' |
| SLC22A6 | 5' -ACC CTC CGC CAC CTC TTC C- 3' | 5' -GGC AGG CAG GTC CAC AGC- 3' |
| CHRM1 | 5' -CCG CTA CTT CTC CGT GAC TC- 3' | 5' -GTG CTC GGT TCT CTG TCT CC- 3' |
| TRPV1 | 5' -GCC CAT GGG GAC TTC TTT A- 3' | 5' -TTC CCT TCT TGT TGG TGA GC- 3' |
| CARKL | 5' -AAT GGA CAG AGG GAG GGA TT- 3' | 5' -TAC GTT CCA GCT TTG GCT CT- 3' |
| TRPV3 | 5' -GAG CCT GTC CAG GAA GTT CA- 3' | 5' -GTG CTT GGC AAA CTT CTT CC- 3' |
| MAFG | 5' -GAG AAG CTG GCC TCA GAG AA- 3' | 5' -GGC ATC CGT CTT GGA CTT TA- 3' |
| PYCR1 | 5' -ACA CCC CAC AAC AAG GAG AC- 3' | 5' -CTG GAG TGT TGG TCA TGC AG- 3' |
| SIRT7 | 5' -GGA CCT GGT AAC GGA GCT G- 3' | 5' -CGC CTG TGT AGA CGA CCA AG- 3' |
| GSTT2 | 5' -CAA TGG CTG GAG GAC AAG TT- 3' | 5' -CCT GAT AGG CCT CTG GTG AG- 3' |
| DDT | 5' -CTG GAG CTG GAC ACG AAT TT- 3' | 5' -GGC TAG CTC CTT GGT GAG AA- 3' |
| SYNGR1 | 5' -TCT GCA TCT ACA ACC GCA AC- 3' | 5' -TTC AGT GGG TTG TCC TTG G- 3' |
| MAP3K7IP1 | 5’ -CCA AGC TGG ACA GAT GAC CT- 3’ | 5’ -CCA CGA AGT TGG TCA CTC G- 3’ |
| CRELD2 | 5' -GGA GAT GGG AGC AGA CAG G- 3' | 5' -ACC CAG CCC ACT TCA CAC T- 3' |
| ALG12 | 5’ -GCG TGA TTT TTG GAC TCT GG- 3’ | 5’ -GAA CAC GAT GAT GGC GAA G-3’ |
| 18S rRNA | 5' -CCG CAG CTA GGA ATA ATG GA- 3' | 5' -CGG TCC AAG AAT TTC ACC TC- 3' |
| AR | 5’ -CTG GAC ACG ACA ACA ACC AG- 3’ | 5’ -CAG ATC AGG GGC GAA GTA GA- 3’ |

**D) siRNA Oligonucleotides**

| **Target** | **Forward Primer** | **Reverse Primer** |
| --- | --- | --- |
|  |  |  |
| AR | 5’-ACG UUU ACU UAU CUU AUG CTT-3’ | 5’-GCA UAA GAU AAG UAA ACG UTT-3’ |
| Non specific | 5’-AAU UUU ACU CGC UCG AUU UTT-3’ | 5’-AAA UCG AGC GAG UAA AAU UTT- 3’ |
