# Supplementary material for: Identification of novel androgen receptor target genes in prostate cancer
Source: Mol Cancer. 2007 Jun 6;6:39. doi: 10.1186/1476-4598-6-39 (PMC1904239; doi:10.1186/1476-4598-6-39)
Supplement: Additional file 2 — Microsatellite analysis in LNCaP and C4-2B. This file provides the results of microsalletile analysis of C4-2B cells versus the parental LNCaP cells. [file 1476-4598-6-39-S2.doc]

| **Locus Designation** | **Chromosome Location** | **Common Sequence Motif** | **Size Range** | **LNCaP** | **C4-2B** |
| --- | --- | --- | --- | --- | --- |
| D3S1358 | 3p | TCTA (TCTG)_1-3_ (TCTA)_n_ | 114-142 | 130/130 | 126/130 |
| vWA | 12p12-pter | TCTA (TCTG)_3-4_ (TCTA)_n_ | 157-197 | 177/185 | 177/185 |
| FGA | 4q28 | (TTTC)_3_ TTTT TTCT (CTTT)_n_ CTCC (TTCC)_2_ | 219-267 | 223/227 | 223/227 |
| Amelogenin | X: p22.1-22.3 |  | 107 | 107 | 107 |
|  | Y: p11.2 |  | 113 | 113 |  |
| D8S1179 | 8 | (TCTR)_n_ | 128-168 | 140/148 | 140/148 |
| D21S11 | 21 | (TCTA)_n_ (TCTG)_n_ [(TCTA)_3_ TA (TCTA)_3_ TCA (TCTA)_2_ TCCA TA] (TCTA)_n_ | 189-243 | 203/219 | 203/207/219* |
| D18S51 | 18q21.3 | (AGAA)_n_ | 273-341 | 281/285 | 281/285 |
| D5S818 | 5q21-31 | (AGAT)_n_ | 135-171 | 147/151 | 147/151 |
| D13S317 | 13q22-31 | (GATA)_n_ | 206-234 | 214/222 | 214/222 |
| D7S820 | 7q11.21-22 | (GATA)_n_ | 258-294 | 270/278 | 270/278 |

**Additional File 2**

**Table 2: Microsatellite analysis in LNCaP and C4-2B**. Genetic profiling was performed using a discriminating system for human identification (AmpFLSTR Profiler Plus^TM^ PCR Amplification Kit, Applied Biosystems Inc.), which amplifies nine tetranucleotide short tandem repeat (STR) loci (D3S1358, D5S818, D8S1179, D13S317, D18S51, D21S11, FGA, and vWA) and the Amelogenin locus (which discriminates different product lengths from the X and Y chromosomes). DNA was extracted from cells growing at log-phase and PCR reactions performed in accordance with the manufacturer’s instructions. 0.8μl of each amplification product was diluted with 10μl formamide containing a fluorescent ROX-labeled size standard (GS-500; Applied Biosystems) at a 20:1 ratio. The diluted products were run on an ABI 3700 Capillary Electrophoresis DNA Analyser (Applied Biosystems) and analyzed with GeneScan v3.5 software (Applied Biosystems). Eighteen of the twenty alleles were identical in the two cell lines and in one case (D21S11) an extra allele was detected in C4-2B cells, indicating that the two lines are mostly isogenic.
